# PPTS Inhibits the TGF-*β*1-Induced Epithelial-Mesenchymal Transition in Human Colorectal Cancer SW480 Cells

**DOI:** 10.1155/2019/2683534

**Published:** 2019-09-22

**Authors:** Ling Zhang, Abid Naeem, Shaofeng Wei, Zexie Li, Zhenzhong Zang, Meng Wang, Yali Liu, Dan Su

**Affiliations:** ^1^Jiangxi University of Traditional Chinese Medicine, 818 Meiling Road, Nanchang 330006, China; ^2^Science and Technology College, Jiangxi University of Traditional Chinese Medicine, 818 Meiling Road, Nanchang 330006, China

## Abstract

The current study investigates the inhibitory effects of Pulsatilla pentacyclic triterpenoid saponins extract (PPTS) on epithelial-mesenchymal transition (EMT) triggered by the transforming growth factor-*β*1 (TGF-*β*1) in human colorectal cancer SW480 cell line, further illustrates the possible mechanism of PPTS inhibition of growth and invasion from the perspective of EMT, and provides new theoretical support for the treatment of tumor by Chinese medicine. The SW480 cells were treated in groups: blank control, TGF-*β*1 (10 ng/mL), and varying concentrations of PPTS cotreated with TGF-*β*1-induced (10 ng/mL) groups. CCK8 was used to detect cell viability; transwell was applied to detect invasion ability, cell migration ability was also determined, ELISA and RT-qPCR were utilized for the determination of CYP3A, CYP2C9, CYP2C19, N-cadherin, and MMP-9 expression. Flow cytometry detection was applied to detect cell cycle and apoptosis. The results obtained have shown that PPTS can significantly inhibit the invasion and migration of tumors in SW480 cells and can also block the S phase in the cell cycle but may produce cytotoxicity in higher doses. The present research work provides substantial evidence that PPTS has a significant inhibitory effect on TGF-*β*1-induced EMT in SW480 cells and it also promotes apoptosis.

## 1. Introduction

Colorectal cancer (CRC) is a standout amongst the most occurring tumors worldwide, and lately, it is the most general reason behind malignancy-related deaths in China and the USA [1, 2]. Despite improvements in the diagnostic and therapeutic modalities, the incidence of colorectal cancer is continuously increasing because of poor prognosis in patients having distantly metastasized tumors and also some of the underlying molecular mechanisms of metastasis are not very clear [3, 4]. Historically, metastasized tumors are challenging to treat. Thus, it is crucial to know the underlying mechanism of colorectal cancer metastasis and treat it [5].

Metastasis and invasion of tumors are the leading causes of death in cancer patients. EMT mediated by TGF-*β*1 can transform tumor epithelial cells into mesenchymal cells, and the tumor cells are thus invasive and migratory, escaping from a primary tumor, and then metastasize [6–8]. Many of the active ingredients in traditional Chinese medicine have a significant inhibitory effect on TGF-*β*1-induced EMT. For example, saikosaponin, *Patrinia villosa* saponins [9], and resveratrol [10] can inhibit EMT transformation by controlling the expression of epithelial marker E-cadherin and the interstitial markers N-cadherin and vimentin. Therefore, an in-depth study of the role of TGF-*β*1-regulated EMT in tumor invasion and metastasis can provide a basis for clinical treatment of tumor metastasis.

*Pulsatilla chinensis* Regel is a well-known traditional Chinese medicine, better known for its anti-inflammatory activity, and also listed in Chinese pharmacopeia with “heat-clearing, detoxifying, cooling blood, and stopping dysentery” properties [11]. Lately, various studies have revealed that it also possesses anti-umor properties and it can play a crucial part in the treatment of a wide range of tumors [12, 13] and also its mechanism of reducing proliferation of different cancer cells such as HELA, 7721, MKN-45, BGC-823, SW116, LoVo, and CaEs-17 cells [14]. According to the previous results [15], as reported by Mi Kwon et al., among the different types of saponins separated from *P koreana*, Pulsatilla saponin D has proved to be a decent contender as a natural agent for the treatment of colon malignancy by managing the AKT/mTOR signaling pathway [16]. Luo also observed that *Pulsatilla* saponin produces a strong antitumor effect in vitro by inhibiting the proliferation of HT29 colon cancer cells and also the possible mechanism of apoptosis [17]. Therefore, *Pulsatilla* pentacyclic triterpenoid saponins (PPTS) extracts have been confirmed to have unequivocally antitumor activity, especially anticolorectal activity.

Although there has been a lot of work done on *Pulsatilla* saponins and their antitumor effect, however, according to our insights, there are no reports available which have shown the effects of PPTS on EMT induced by TGF-*β*1 in human colorectal SW480 cancer cells. In this work, SW480 cells were treated with different concentrations of PPTS, in order to identify tumor invasion and EMT-related proteins, with a plan to preliminarily uncover the impacts of PPTS inhibiting TGF-*β*1-induced EMT and its primary mechanism, and also provide the basis for additional investigations.

## 2. Materials and Methods

SW480 cells were obtained from the Chinese Academy of Sciences SUER0200 (XR). RPMI-1640 culture media (1×) was provided by KGI bio-KGM41500S-500; trypsin-EDTA digestive juice, crystal violet staining solution and Annexin V-FITC/PI, and apoptosis kit were purchased from Solarbio T1300. PBS was obtained from BI 02-024-1ACS, and OPTI-MEM® I (1×) was purchased from gibco 331985-062. TGF-*β*1 was provided by Beijing Boaosen Biotechnology Co., Ltd. AG12051847, CCK8 cell proliferation detection reagent was obtained from KGI Bio-KGA317, and PPTS was extracted from the plant Pulsatilla [18].

### 2.1. Experimentation Group and Cell Culture

The whole study was designed and divided into five groups. All the experiments were conducted in a pathogen-free environment. Group (*A*) is the blank control group (culture solution), (*B*) TGF-*β*1 (10 ng/mL) induction group, (*C*) PPTS low dose (5 *μ*g/mL) + TGF-*β*1 (10 ng/mL) induction group, (*D*) PPTS medium dose (10 *μ*g/mL) + TGF-*β*1 (10 ng/mL) induction group, and (*E*) PPTS high dose (20 *μ*g/mL) + TGF-*β*1 (10 ng/mL) induction group.

The SW480 human colorectal cancer cell line was routinely cultured in RPMI-1640 medium completed with 10% calf serum under the environment of 5% CO_2_ and 37°C, and cells in the logarithmic growth stage were chosen for cell experiments.

### 2.2. Cell Counting Kit-8 Test

Cell Counting Kit-8 (M*μ*LTI SCIENCES Associated Bio-CCS102) was chosen to determine the rate of cell proliferation by following the instructions of the manufacturer. Briefly, SW480 cells were seeded in 96-well plates at a density of 5 × 10^3^ cells/well. On the following day, the medium was replaced with 100 *μ*L fresh medium containing different concentrations of PPTS (in correspondence to the group) and incubated for 2 days. The medium was then disposed of, and CCK-8 reagent was added into the cells and then incubated for 4 hours. The absorbance was recorded at 450 nm utilizing a microplate reader (Rayto, RT-6100). All the experiments were performed in triplicate.

### 2.3. Cell Cycle Detection by Flow Cytometry

The passage cell suspension was diluted to a density of 1 × 10^5^/well and added to 6-well plate and then incubated with 5% CO_2_ at 37°C. The liquid exchange treatment was performed after the cells adhered to a density of 80%, and then 2 mL medium containing an appropriate concentration of drugs in correspondence to the group concentrations was added to each well. After 48 h incubation, it was centrifuged at 1500 rpm for 3 min. The supernatant was removed and 1 mL PBS was added, then the suspension was centrifuged again at 1500 rpm for another 3 min, and the supernatant was discarded. Then, 1 mL DNA staining solution and 10 *μ*L permeabilization solution were added and vortexed for 5–10 seconds to mix it properly and finally incubated at 25°C for 30 min in the darkness. The data were analyzed using a flow cytometer (NovoCyteTM).

### 2.4. Apoptosis Detection by Flow Cytometry

The SW480 cells were seeded into a 6-well plate at a density of 1 × 10^5^/well and placed in an incubator with the conditions set at 37°C temperature with 5% CO_2_. When the cells adhered to a density of 80%, 2 mL of medium was added to each well at a drug concentration by the groups. After 48 hrs, cells were collected and centrifuged and the supernatant was removed. Further adding 1 mL PBS, the suspension was centrifuged again at 1500 rpm for another 3 min followed by the removal of the supernatant, and cells were resuspended in precooled 1 × binding buffer. Then, 3 *μ*L of Annexin V-FITC and 5 *μ*L of PI-PE were added to each tube with gentle mixing and incubated in the dark for 10 min at 25°C. Finally, 200 *μ*L of precooled 1 × binding buffer was added to every tube, mixed well, and measured by a flow cytometer.

### 2.5. Cell Invasion Assay

For the determination of Transwell assay in SW480 cells, the cells were harvested and seeded into 24-well Transwell plate (Sigma-Aldrich) at the density of 5 × 10^4^/well. After sometime, the cells were washed with PBS for 5 minutes and treated with 0.1% crystal violet for 1 h. Then, the cells in the chamber were cleared off with cotton, and the chamber was inverted and placed on a glass slide for a photograph. After that, the staining liquid was removed from the well, and the dye solution was dissolved in 2 mL of 33% acetic acid in each well, thoroughly mixed and allowed to stand, and measured by using an ultraviolet spectrophotometer at a wavelength of 570 nm.

### 2.6. Cell Migration Assay

The cells were seeded at a density of 5 × 10^4^/well into the 12-well plates in a well-controlled environment for scratch migration. The ruler was used to draw two parallel lines at the bottom of the plates. After trypsinization, the cells were centrifuged, followed by disposition of the supernatant. The pellet of cells was resuspended in the medium before spreading it on the orifice plate and then placed in an incubator having suitable conditions for cultivation. After reaching a density of 100%, each well was scratched with a pipette tip. The medium was discarded and washed with PBS, and then serum-free medium was added. Then, a photograph was taken where the scratches were straightened, and a photo of each well at 0 h was also taken. The scratched culture plate was placed in the incubator for cultivation; after 24 h, the scratches of each well were photographed again and the same photographing position was used as it was in the case of 0 h (fixed point), that is, the same position is taken at two-time points. The rate of cell migration was calculated by analyzing the corresponding scratch width data of 48 h, 24 h, and 0 h.

### 2.7. RT-qPCR Detection

The real-time quantitative polymerase chain reaction was performed by extracting RNA from the cultured cells using Trizol Reagent (CWBIO Kang Wei Century CW0580S); its purity and concentration were also determined by using the uLtrapure RNA extraction kit (CWBIO Kang Wei Century CW0581M). The cDNA was synthesized by using the reverse transcription of the HiFiScript cDNA first-strand synthesis kit (CWBIO Kang Wei Century CW2569M).

The reverse transcription system (Table 1) was vortexed to mix appropriately, centrifuged, and collected the solution at the bottom of the tube. After adding ①, ②, and ③, the solution was incubated at 70°C for 10 min and ice bathed quickly for 2 min. Then, ④, ⑤, and ⑥ were added and firstly incubated at 50°C for 15 min and then at 85°C for 5 min. After the complete reaction, these were briefly centrifuged and placed at −80°C in a refrigerator in order to prevent degradation. Primers information is included in Table 2.

### 2.8. ELISA Test

ELISA was employed to determine the changes in protein expression. The ELISA assay was performed according to the instructions in the kit. Briefly, the blank and sample wells were set correctly after the addition of 50 *μ*L of the standard to each well. Then, 40 *μ*L sample dilution was added to the sample well and 10 *μ*L sample to the enzyme-labeled plate. Next, 100 *μ*L of the enzyme-labeled reagent was added to each well except the blank wells and then incubated. After washing and drying, 50 *μ*L developer A and B were added to each well and adequately mixed. Finally, the reaction was terminated by the addition of 50 *μ*L stopping solution, and the OD value of each well was estimated at 450 nm.

## 3. Statistical Analysis

The whole data were analyzed using GraphPad Prism software version 7 (GraphPad Prism, Inc., San Diego, CA, USA). One-way ANOVA (Dunnett's) and two-way ANOVA (Bonferroni's) analyses were used for comparisons. The results were stated as $\bar{x}\pm s$. The significant difference between the groups was set at (*P* < 0.05).

## 4. Results

### 4.1. CCK8 Detection

**Figure 1:**
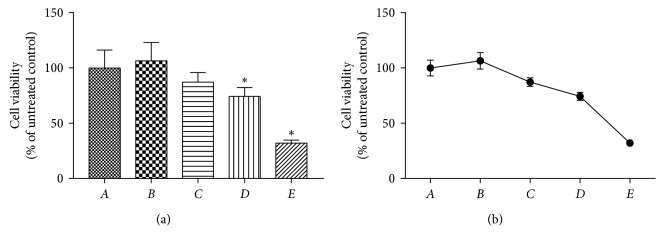
The survival rate of SW480 in each group after 48 hours by CCK8 detection (compared to the blank control group, ^*∗*^*P* < 0.05. Group *A* is the blank control group, *B* is the TGF-*β*1 (10 ng/ml) induction group, *C* is the PPTS low-dose (5 *μ*g/mL) + TGF-*β*1 (10 ng/ml) induction group, *D* is the PPTS medium-dose (10 *μ*g/mL) + TGF-*β*1 (10 ng/ml) induction group, and *E* is the PPTS high-dose (20 *μ*g/mL) + TGF-*β*1 (10 ng/ml) induction group).

The cytotoxic effect of PPTS was determined on colorectal cancer SW480 cells utilizing CCK-8 detection. SW480 cells were treated with varying concentrations of PPTS (5, 10, and 20 *μ*g/mL) for 48 h. The results depicted in (Figure 1) show that PPTS inhibited the expansion of SW480 cells in a concentration-dependent way. The cell expansion was restrained at all concentrations of PPTS, but the impact of a high dose of PPTS was more significant than others in SW480 cells (*P* < 0.05).

### 4.2. Cell Cycle Detection by Flow Cytometry

**Figure 2:**
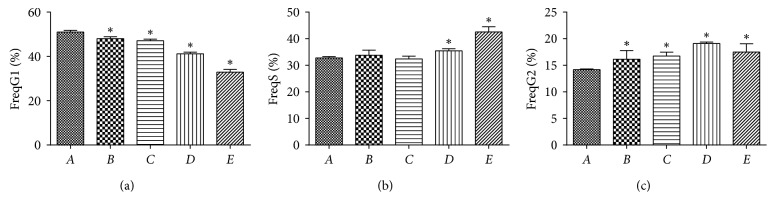
The proportion of SW480 in G1, S, and G2 phases after 48 h by flow cytometry detection (compared with the blank control group, ^*∗*^*P* < 0.05).

The cell cycle was determined after 48 hours of cell incubation (treated with PPTS) by using flow cytometry. The results exhibited (Figure 2) that the proportion of SW480 cells decreased in G1 phase and increased in S and G2 phases after the action of PPTS as compared to the blank control group and induction group. In the blank control group, proportion of SW480 cells is G1  > S > G2; in the induction group, the ratio of SW480 cells is G1  > S > G2; and in PPTS high-dose + TPF-*β*1 induction group, the proportion of SW480 cells was like S > G1 > G2, which indicates that PPTS may block the S phase.

### 4.3. Apoptosis by Flow Cytometry

**Figure 3:**
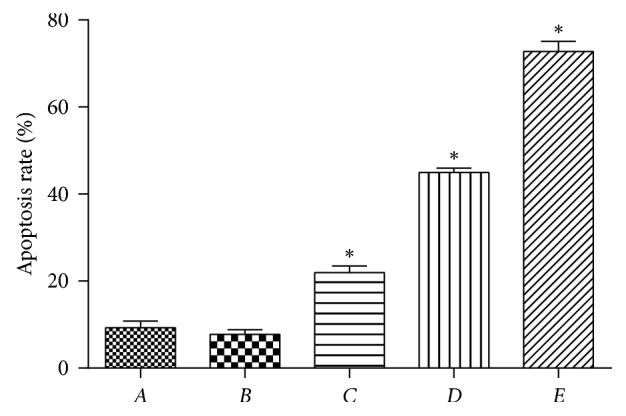
Apoptotic rate of SW480 in each group after flow-cycle detection for 48 h (^*∗*^*P* < 0.05 compared with the blank control group).

The CCK8 determination was carried out after 48 h of incubation. The results depict that PPTS could elevate the apoptosis of SW480 cells under the action of TGF-*β*1 in comparison to the blank control group and induction group. Apoptotic rate of SW480 cells was directly proportional to the concentration of PPTS; as the concentration increased, the apoptotic rate also increased. The apoptotic rate was highest in the high-dose PPTS + TGF-*β*1 induction group, as shown in (Figure 3).

### 4.4. Cell Invasion Assay

**Figure 4:**
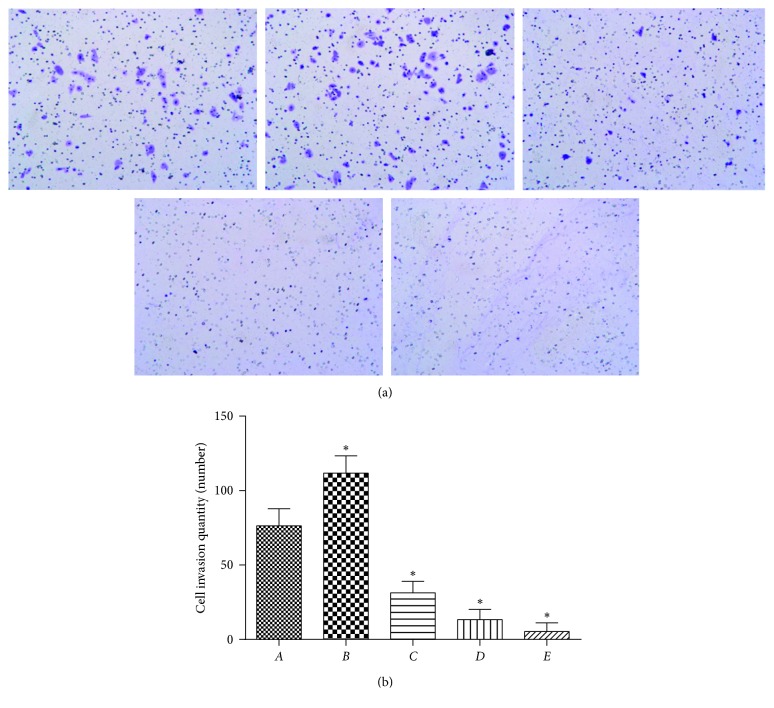
Effect of PPTS on TGF-*β*1-induced invasion of SW480 cells by the transwell test.

The impacts of PPTS on cell invasion were determined by the transwell invasion assay. We observed that when treated with 10 ng/mL of TGF-*β*1, motility and invasive capacity of SW480 cells amplified (Figure 4). However, PPTS could restrict the invasion and migration of SW480 cells in a dose-dependent manner, which were induced by TGF-*β*1 (number of cells in *B* > *C* > *D* > *E*, while A is a control group).

### 4.5. Cell Scratch Migration

**Figure 5:**
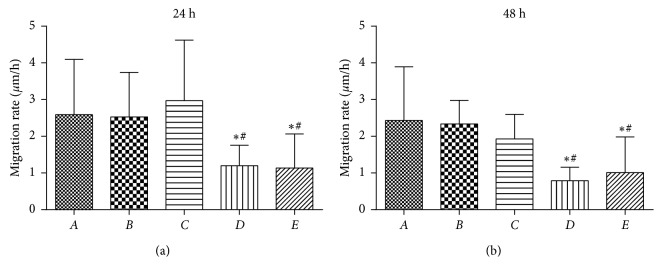
The migration rate of SW480 cells after 24 h and 48 h by the cell scratching assay (^*∗*^*P* < 0.05 compared with the blank control group; ^#^*P* < 0.05 compared with the TGF-*β*1 induction group).

The scratch migration ability of SW480 cells was analyzed and compared after treatment with different concentrations of PPTS for 24 h and 48 h periods. It showed that there was no significant difference between TGF-*β*1 induction group and blank control group, but the addition of PPTS had a significant effect on cell migration ability and especially by the high concentration of PPTS which significantly weakened the cell migration ability as shown in Figure 5.

### 4.6. RT-qPCR Detection

The results of RNA purity were obtained, and the ratio of OD260/OD280 measured by each group was between 1.9 and 2.0, indicating that the quality of RNA extracted in the early stage of the experiment was acceptable and the purity was up to the standard (Table 3). The contamination of protein, DNA, and other impurities was excluded, and the study was in line with the requirements of subsequent experiments.

**Figure 6:**
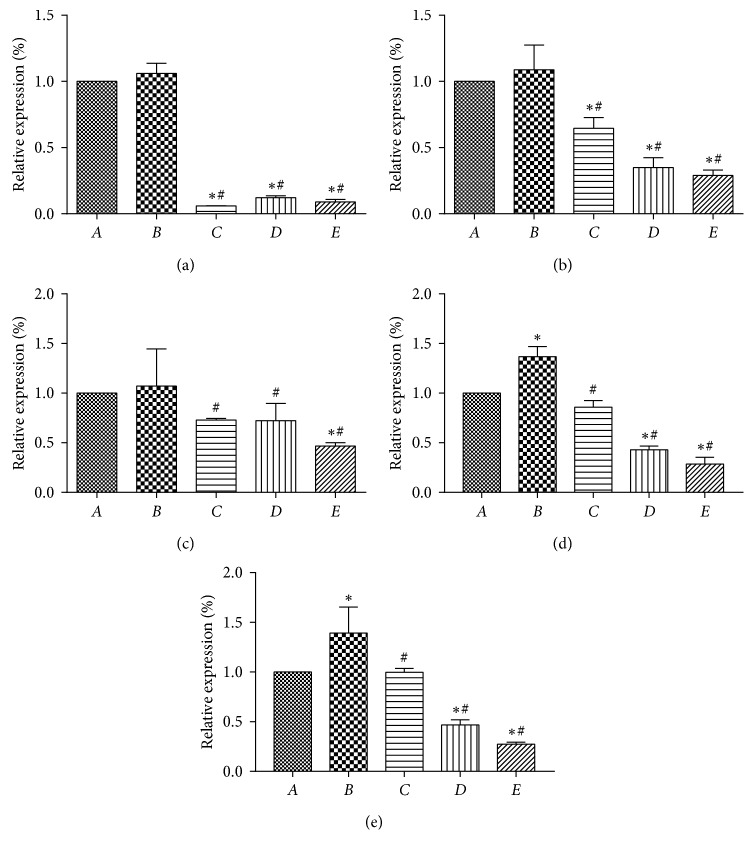
The expression of (a) MMP-9, (b) CYP2C9, (c) CYP3A, (d) N-cadherin, and (e) CYP2C19 was detected by real-time qPCR (^*∗*^*P* < 0.05 compared with the blank control group; ^#^*P* < 0.05 compared with the TGF-*β*1 induction group).

The amplification curves of the gene fragment were amplified by the five genes: MMP-9, CYP2C9, CYP3A, N-cadherin, and CYP2C19. The GAPDH primers were used to designate the consistency of amplification and plateau. PPTS were able to alter the expression level of invasive genes associated with SW480 cells instigated by TGF-*β*1. The results showed (Figure 6) that the relative expression of MMP-9, CYP2C9, CYP3A, N-cadherin, and CYP2C19 was elevated in SW480 cells after 48 h of treatment with TGF-*β*1. In comparison to the single TGF-*β*1 induction group, the expression of all MMP-9, CYP2C9, CYP3A, N-cadherin, and CYP2C19 were decreased with the treatment of PPTS. It was demonstrated at the molecular level that PPTS has an inhibitory effect on the process of EMT and may reduce the invasive and migrative abilities of human intestinal cancer SW480 cells by inhibiting the EMT process.

### 4.7. ELISA Test

**Figure 7:**
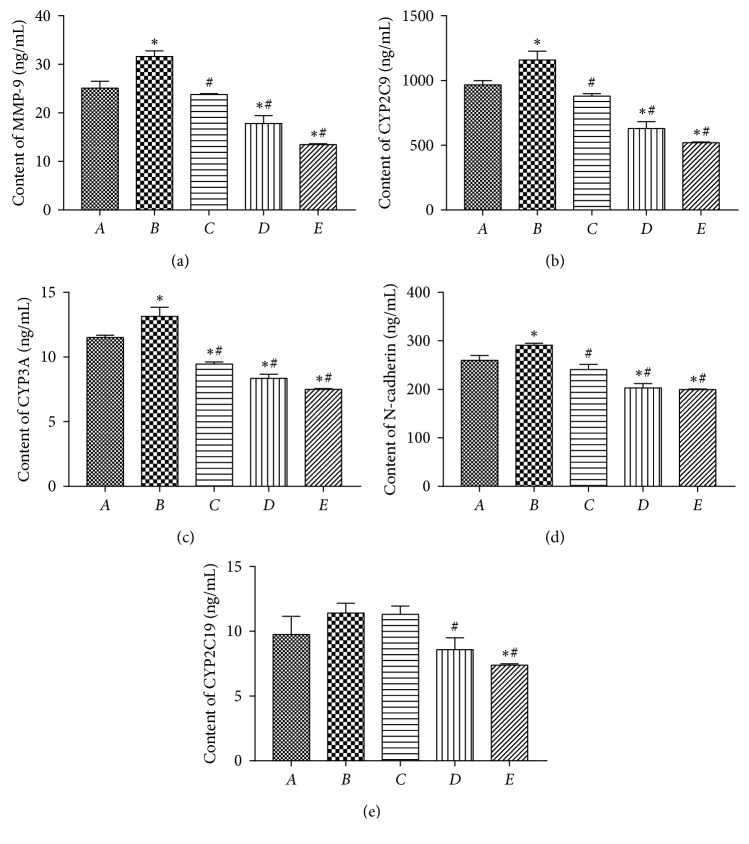
The content of (a) MMP-9, (b) CYP2C9, (c) CYP3A, (d) N-cadherin, and (e) CYP2C19 detected by ELISA (^*∗*^*P* < 0.05 compared with the blank control group).

The ELISA results are shown in Figure 7, which indicates that the contents of MMP-9, CYP2C9, CYP3A, N-cadherin, and CYP2C19 in tissues were determined. When the results are compared to the blank control group, the relative expression of MMP-9, CYP2C9, CYP3A, N-cadherin, and CYP2C19 in the TGF-*β*1 induction group increased with significant differences except CYP2C19 compared to the blank control group. When compared to the single TGF-*β*1 induction group, the expression of MMP-9, CYP2C9, CYP2C19, CYP3A, and N-cadherin was significantly decreased in the PPTS + induction group.

## 5. Discussion

EMT plays a crucial part in embryonic and tumor growth, which helps in transformation of the epithelial cell into mesenchymal cell phenotype having mesenchymal attributes, therefore offering to ascend decreased intercellular attachment, disappeared cell polarity, and improved motility and migration [8, 19, 20]. Along these lines, the API that can block or inverse EMT may turn into a novel chemotherapeutic for antitumor invasion therapy [21].

Pulsatilla was first published in “Shen Nong's Herbal Classic.” Body temperature rises during conditions such as malaria, cold sore, and phlegm, relieving blood clot pain and other accumulated symptoms. Among these, “symptoms accumulate” refers to the category of tumors in modern medicine, which shows that, in ancient times, *Pulsatilla* was used to treat diseases like tumors [22].

The results of cell phenotype experiments revealed that PPTS had an inhibitory effect in SW480 cells in a concentration-dependent way similar to the classic antineoplastic drugs which are already in the market, such as cyclophosphamide, cytarabine, fluorouracil, and platinum-based drugs [23]. The S phase block during the cell cycle indicated that DNA molecular replication and cell division were significantly affected. Moreover, the TGF-*β*1 signaling pathway was employed to induce EMT.

It has been recently demonstrated that N-cadherin expression is related to tumor growth, differentiation, growth size, nodes, and metastasis phase, proposing a possible role of N-cadherin in colorectal cancer progression, such as Su et al. [24] found that impedance with N-cadherin expression by a monoclonal immune response can viably delay the survival in an unconstrained exceptionally metastatic pancreatic cancer model [25]. It has been additionally proved in a prostate cancer cell line that overexpression of N-cadherin increases tumor development, intrusion, and metastasis through EMT [26]. After silencing of N-cadherin, the proliferative and transitory capacities of HT 29 cells were hindered, demonstrating that N-cadherin may prompt metastatic potency in CRC cells [27].

N-cadherin is thought to be a nifty oncoprotein because of its role in tumor development, intrusion, metastasis, and formation of new blood vessels [28]. It has additionally been discovered that N-cadherin expression is adversely connected with E-cadherin expression, inferring a conceivable role of N-cadherin in inducing the EMT in CRC. It has been demonstrated that N-cadherin silencing leads to the upregulation of E-cadherin expression, which was also confirmed by the immunohistochemical investigations [27]. The best-portrayed marker of the EMT is the loss of E-cadherin and upregulation of N-cadherin, which increases the progression of CRC moderately by inducing EMT. This finding is similar to an investigation by Zhang et al. [29], which showed that N-cadherin accelerated the process of proliferation as well as invasion. From this, it can be deduced that downregulation of N-cadherin expression will likewise recuperate the expression of epithelial markers, for example, E-cadherin, and results in the inversion of EMT. In our current study, we have shown that PPTS can inhibit TGF-*β*1-induced EMT in SW480 colon cancer cells by downregulating the expression of N-cadherin. Most importantly, PPTS can also inverse epithelial cell phenotype for TGF-*β*1-induced mesenchymal transition by recovering the expression of epithelial markers [9].

The four significant cancer hallmarks, such as invasion, migration, metastasis, and neovascularization, are influenced by the surrounding microenvironment of the cells [30]. Overexpression of MMP-9 and various others has been related to epithelial-mesenchymal transition; therefore, it was chosen for this study instead of other commonly used proteins [31]. For growth and migration, the cancer cells need to develop new blood vessels. The initial phase is to degrade the physical boundaries and numerous other macromolecules of ECM; in this way, adjusting cell-cell and cell-extracellular matrix relations promotes cell invasion and also produces proangiogenic factors [32]. So, MMP-9 takes part in angiogenic switch since it results in increasing the critical factors required in this procedure, for example, the vascular endothelial growth factor (VEGF), the most potent mediator of tumor vasculature and basic fibroblast growth factor (bFGF), by degrading the extracellular segments, for example, collagen types IV, XVIII, and perlecan, respectively [30, 33]. Cellular movement is exceedingly identified with the proteolytic action of MMPs and ADAMs, directing the dynamic cell-cell and ECM-cell relation amid migration [34]. Overexpression of MMP-9 and other MMPs results in the suppression of T-lymphocyte proliferation and response against tumor affected cells, since they release TGF-*β*, a noteworthy silencer of T-lymphocyte response against malignant cells [35]. In this study, we have found that PPTS can decrease the expression of MMP-9, which would decline malignant cell sensation to NK cells by producing a small bioactive portion from the a1-proteinase inhibitor [36].

The steroid hydroxylase cytochrome P450 has attracted much attention over the years due to its multiple roles for essential events in cellular physiology [21, 37], such as CYP1B1 can enhance cell expansion and metastasis by inducing EMT and stimulation of Wnt/*β*-catenin signaling via Sp1 upregulation [38]. The CYP4Z1 30UTR could limit migration and EMT of breast cancer cells acting as a ceRNA for E-cadherin [39], and MicroRNA-17 induces EMT persistent with the cancer stem cell phenotype by controlling CYP7B1 expression in colon cancer [40]. Moreover, the CYP3A4 level is correlated by WT1 gene expression and is associated with a weaker response to taxane treatment. CYP2C9 has been associated with a growing risk of colorectal cancer [41]. Both CYP2C9 and CYP2C19 expressions are found to be critical in colorectal cancer [37]. Therefore, in the present research work, CYP2C9, CYP3A, and CYP2C19 were selected to illustrate the effect of the CYP450 system on the progression of colorectal cancer [41]. As presumed, we discovered through further investigation that the CYP450 signaling pathway plays an integral part in TGF-*β*1-induced EMT in SW480 cell. It indicated PPTS would specifically antagonize related metabolites, thereby disturbing the biosynthesis of nucleic acids, especially DNA, and the division and reproduction of histiocytes.

Additionally, the effects of PPTS on other pathways induced by TGF-*β*1 remain to be determined. Thus, our findings warrant further evaluations of PPTS's in vitro and in vivo functions as well as its clinical utility in the treatment of diseases.

## 6. Conclusion

In summary, as a traditional anticancer herb's active ingredient, PPTS hindered TGF-*β*1-induced EMT and diminished migration and invasion in SW480 cells, and it also promotes apoptosis as demonstrated by the declination in the expression of MMP-9, CYP2C9, CYP2C19, CYP3A, and N-cadherin. We observed that the TGF-*β*1-induction group increased the migrative and invasive capacities of SW480 cells; however, PPTS could hinder the advancing impact. Consequently, PPTS may restrain the migration and metastasis of CRC cells by stifling TGF-*β*1-induced EMT. Furthermore, CYP450 signaling pathways also play an essential part in the TGF-*β*1-induced EMT in SW480 cells.

## Figures and Tables

**Table 1:** Reverse transcription system.

Reagent	20 *μ*L reaction system
dNTP mix	4* μ*L①
Primer mix	2* μ*L②
RNA template	7* μ*L③
5x RT buffer	4* μ*L④
DTT	2* μ*L⑤
HiFiScript	1* μ*L⑥

**Table 2:** Primer information.

Primer name	Primer sequence	Primer length (bp)	Product length (bp)	Annealing temperature (°C)
MMP-9F	TTGACAGCGACAAGAAGTGG	20	144	57.9
MMP-9R	CAGTGAAGCGGTACATAGGG	20		
CYP2C9 F	GCCTTTTCTCACCTGTCATCTCAC	24	137	60.5
CYP2C9 R	CAATGCAACTGTTACAGAGTATGGA	25		
CYP3A F	CACAGATCCCCCTGAAATTAAGCTA	26	106	61.2
CYP3A R	AAAATTCAGGCTCCACTTACGGTG	24		
N-cadherin F	TCTGGGTCTGTTTTATTACTCCTGG	25	71	60
N-cadherin R	GCGAGCTGATGACAAATAGCG	21		
CYP2C19F	ATTGAATGAAAACATCAGGATTG	23	182	55
CYP2C19R	GAGGGTTGTTGATGTCCATC	20		
GAPDH F	GAGTCAACGGATTTGGTCG	19	215	56.5
GAPDH R	CTGGAAGATGGTGATGGGAT	20		

*Note.* Primer synthesis company: Bioengineering Biotechnology (Shanghai) Co. Ltd., China.

**Table 3:** RNA concentration purity.

Sample name	Sample RNA concentration (ng/*μ*L)	Sample RNA purity
Blank control group	686.4	2.00
TGF-*β*1 (10 ng/ml) induction group	698.8	1.98
PPTS low-dose (5 *μ*g/mL) + TGF-*β*1 (10 ng/ml) induction group	565.2	2.00
PPTS medium-dose (10 *μ*g/mL) + TGF-*β*1 (10 ng/ml) induction group	382.4	1.95
PPTS high-dose (20 *μ*g/mL) + TGF-*β*1 (10 ng/ml) induction group	102.4	1.97

## Data Availability

The data used to support the findings of this study are available from the corresponding author upon request.
